# Impact of obesity on the response to tumor necrosis factor inhibitors in axial spondyloarthritis

**DOI:** 10.1186/s13075-017-1372-3

**Published:** 2017-07-19

**Authors:** Raphael Micheroli, Monika Hebeisen, Lukas M. Wildi, Pascale Exer, Giorgio Tamborrini, Jürg Bernhard, Burkhard Möller, Pascal Zufferey, Michael J. Nissen, Almut Scherer, Adrian Ciurea, on behalf of the Rheumatologists of the Swiss Clinical Quality Management Program

**Affiliations:** 1 Department of Rheumatology, Zurich University Hospital, Gloriastrasse 25, CH-8091 Zurich, Switzerland; 2 Swiss Clinical Quality Management Foundation, Zurich, Switzerland; 3 Praxis Rheuma-Basel, Basel, Switzerland; 4 Ultraschall Zentrum Rheumatologie, Basel, Switzerland; 5 Department of Rheumatology and Rehabilitation, Bürgerspital, Solothurn, Switzerland; 6 Department of Rheumatology, Allergology and Clinical Immunology, Inselspital, Bern, Switzerland; 7 Department of Rheumatology, CHUV, Lausanne, Switzerland; 8 Department of Rheumatology, University Hospital, Geneva, Switzerland

**Keywords:** Axial spondyloarthritis, Ankylosing spondylitis, Obesity, TNF inhibition

## Abstract

**Background:**

Few studies have investigated the impact of obesity on the response to tumor necrosis factor inhibitors (TNFi) in patients with axial spondyloarthritis (axSpA). The aim of our study was to investigate the impact of different body mass index (BMI) categories on TNFi response in a large cohort of patients with axSpA.

**Methods:**

Patients with axSpA within the Swiss Clinical Quality Management (SCQM) program were included in the current study if they fulfilled the Assessment in Spondyloarthritis International Society (ASAS) criteria for axSpA, started a first TNFi after recruitment, and had available BMI data as well as a baseline and follow-up visit at 1 year (±6 months). Patients were categorized according to BMI: normal (BMI 18.5 to <25), overweight (BMI 25–30), and obese (BMI >30). We evaluated the proportion of patients achieving the 40% improvement in ASAS criteria (ASAS40), as well as Ankylosing Spondylitis Disease Activity Score (ASDAS) improvement and status scores at 1 year. Patients having discontinued the TNFi were considered nonresponders. We controlled for age, sex, HLA-B27, axSpA type, BASDAI, BASMI, elevated C-reactive protein (CRP), current smoking, enthesitis, physical exercise, and co-medication with disease-modifying antirheumatic drugs, as well as with nonsteroidal anti-inflammatory drugs in multiple adjusted logistic regression analyses.

**Results:**

A total of 624 axSpA patients starting a first TNFi were considered in the current study (332 patients of normal weight, 204 patients with overweight, and 88 obese patients). Obese individuals were older, had higher BASDAI levels, and had a more important impairment of physical function in comparison to patients of normal weight, while ASDAS and CRP levels were comparable between the three BMI groups. An ASAS40 response was reached by 44%, 34%, and 29% of patients of normal weight, overweight, and obesity, respectively (overall *p* = 0.02). Significantly lower odds ratios (ORs) for achieving ASAS40 response were found in adjusted analyses in obese patients versus patients with normal BMI (OR 0.27, 95% confidence interval (CI) 0.09–0.70). The respective adjusted ASAS40 OR in overweight versus normal weight patients was 0.62 (95% CI 0.24–1.14). Comparable results were found for the other outcomes assessed.

**Conclusions:**

Obesity is associated with significantly lower response rates to TNFi in patients with axSpA.

**Electronic supplementary material:**

The online version of this article (doi:10.1186/s13075-017-1372-3) contains supplementary material, which is available to authorized users.

## Background

While the association of psoriasis and psoriatic arthritis with obesity is established [1, 2], few studies have investigated the issue of obesity in patients with predominantly axial involvement of spondyloarthritis (axSpA), and particularly ankylosing spondylitis (AS). In a small study of 46 patients with AS, an increased body mass index (BMI) was associated with a greater disease activity and functional limitation, as assessed by the Bath Ankylosing Spondylitis Disease Activity and Functional Indices (BASDAI and BASFI, respectively) [3]. These findings were confirmed in a larger cohort of 461 axSpA patients from the Netherlands [4].

Obese patients with psoriatic arthritis have been shown to have a lower probability of obtaining minimal disease activity either in the absence of systemic immunosuppressive treatment or upon treatment with conventional or biologic disease-modifying antirheumatic drugs (DMARDs) [5, 6]. However, with regard to AS patients, the impact of a high BMI on the response to tumor necrosis factor inhibitors (TNFi) has only been formally demonstrated for infliximab in two retrospective studies [7, 8]. The aim of this study was to investigate the impact of BMI on the response to TNFi in a large cohort of patients with axSpA.

## Methods

### Study population

The Swiss Clinical Quality Management (SCQM) rheumatologists established an ongoing cohort of patients diagnosed with axSpA in 2004 [9]. Assessments at baseline and annual visits are performed according to the recommendations of the Assessment of SpondyloArthritis International Society (ASAS) [10]. We included patients in the current study if they fulfilled the 2009 ASAS criteria for axSpA [11], if they had started treatment with a first TNFi after recruitment, and had baseline BMI data (visit allowed 0–3 months before treatment start) and a follow-up visit at 1 year (±6 months). Patients were categorized according to BMI to the following groups: normal (BMI 18.5 to <25), overweight (BMI 25–30), and obese (BMI >30). As measured height is influenced by kyphosis in advanced disease, corrected height values were calculated by means of geometry using the measurements of the occiput-to-wall distance (see Additional file 1: Figure S1 and Additional file 2: Figure S2).

We excluded patients with concurrent fibromyalgia (as indicated by the treating rheumatologist in the comorbidity questionnaire; *n* = 25) and patients with a BMI <18.5 (*n* = 18). The study was approved by the Ethics Commission of the Canton of Zurich. All patients provided written informed consent.

### Response to treatment with a first TNF inhibitor

Response to anti-TNF treatment was assessed at 1 year (±6 months). Patients having discontinued treatment were considered as nonresponders [12]. The primary outcome was the achievement of the 40% improvement in ASAS criteria (ASAS40) [10]. The following additional effectiveness measures were evaluated: the ASAS criteria for partial remission (ASAS-PR), a 50% reduction in the Bath Ankylosing Spondylitis Disease Activity Index (BASDAI-50), the proportion of patients achieving an Ankylosing Spondylitis Disease Activity Score (ASDAS) <2.1 (reflecting moderate disease activity) or ASDAS <1.3 (corresponding to inactive disease), as well as a clinically important improvement in ASDAS (change of ≥1.1 between baseline and follow-up) or a major improvement in ASDAS (change of ≥2.0) [13]. Drug retention was assessed as a further secondary outcome. Treatment courses ongoing at the end of the study were censored at the last visit registered in SCQM.

### Statistical analysis

We compared baseline characteristics between different BMI categories using the Fisher’s exact test for categorical variables and the Mann-Whitney test for continuous variables. The significance of differences in crude response rates at 1 year was assessed using the Fisher’s exact test. Logistic regression analysis was used to estimate an adjusted ratio for ASAS40, ASAS-PR, BASDAI-50, ASDAS clinically important or major improvement, and ASDAS status scores <2.1 and <1.3 with adjustment for the following parameters: age, gender, BASDAI, Bath Ankylosing Spondylitis Mobility Index (BASMI), human leucocyte antigen-B27 (HLA-B27), classification as nonradiographic axSpA (nr-axSpA) vs. AS, elevated C-reactive protein (CRP) status, physical exercise (yes/no), and current smoking. We tested for interaction between the use of infliximab and BMI categories. Drug maintenance was evaluated with Kaplan-Meier plots and log-rank test, as well as with multiple adjusted Cox proportional hazards models to estimate a covariate-adjusted effect of BMI categories. The same covariates were used in this model as in the response analysis (see above). R statistical software was used for all analyses.

## Results

A total of 624 patients had started a first TNFi after recruitment into SCQM and fulfilled the inclusion criteria (normal weight in 332 patients (53%), overweight in 204 patients (33%), and obesity in 88 patients (14%)). Baseline characteristics of these patients are shown in Table 1. Obese individuals were older, had higher BASDAI and Maastricht Ankylosing Spondylitis Enthesitis Score (MASES) levels, and a more important impairment of physical function in comparison to patients of normal weight. There were no significant differences between the BMI categories with regard to the ASDAS, as well as regarding the proportion of patients with elevated CRP, peripheral arthritis, and extraskeletal manifestations. The use of individual biologics as a first TNFi was also similarly distributed. The proportion of patients treated with infliximab, which in contrast to the other anti-TNF agents is dosed in a weight-dependent manner, was similar for the different BMI categories (22%, 20%, and 25% for normal weight, overweight, and obesity, respectively; overall *p* = 0.57). One year after TNFi initiation, mean BASDAI (±SD) decreased from 5.3 ± 2.0 to 2.9 ± 2.2 in normal weight patients, from 5.6 ± 1.9 to 3.2 ± 2.2 in overweight patients, and from 6.1 ± 1.7 to 4.1 ± 2.4 in obese patients. While the intensity of fatigue—assessed by BASDAI question 1—was comparable between the three BMI categories at the start of TNFi (overall *p* = 0.44; Table 1), it was significantly higher in obese patients in comparison to patients of normal weight and who were overweight at the 1-year follow-up visit: mean levels (±SD) 5.0 ± 2.4 vs. 4.0 ± 2.7 vs. 4.1 ± 2.6, respectively; overall *p* = 0.005. With regard to improvement of enthesitis, median MASES (interquartile range (IQR)) decreased from 1 (0–4) to 0 (0–2) in patients with normal BMI, from 2 (0–4) to 0 (0–1) in overweight patients, and from 4 (2–6) to 1 (0–3) in obese patients.

**Table 1:** Baseline characteristics at the start of first TNF inhibitor

Parameter	*n =* 624	All Patients *n* = 624	BMI category		
			Normal *n* = 332	Overweight *n* = 204	Obese *n* = 88
BMI	624	25.4 (4.3)	22.3 (1.7)	27.0 (1.4)*	33.2 (3.2)* ^#^
Male sex, %	624	62.2	55.7	73.5*	60.2
mNYc positive, %	437	73.9	73.1	75.2	74.1
Age, years	624	39.4 (11.6)	37.4 (11.3)	41.1 (11.7)*	43.2 (10.5)*
Symptom duration, years	619	13.0 (10.9)	12.2 (10.3)	13.6 (11.7)	14.5 (11.0)
HLA-B27 positive, %	571	78.1	80.5	77.2	70.9
BASDAI	549	5.5 (1.9)	5.3 (2.0)	5.6 (1.9)	6.1 (1.7)*
BASDAI Question 1	556	6.0 (2.3)	5.8 (2.3)	6.1 (2.2)	6.1 (2.0)
BASDAI Question 2	555	6.7 (2.3)	6.5 (2.4)	6.9 (2.2)	7.0 (2.1)
BASDAI Question 3	553	4.2 (3.0)	3.9 (3.0)	4.4 (3.0)	5.4 (2.8)*
BASDAI Question 4	556	5.2 (3.0)	5.1 (3.1)	5.2 (3.0)	5.8 (2.9)
BASDAI Question 5	554	6.1 (2.7)	5.9 (2.7)	6.3 (2.5)	6.6 (2.8)
BASDAI Question 6	554	4.9 (2.9)	4.9 (3.0)	4.8 (2.8)	5.3 (2.7)
Patient GA	552	6.4 (2.3)	6.2 (2.5)	6.6 (2.2)	6.6 (2.0)
Physician GA	600	4.9 (1.9)	4.9 (1.8)	4.9 (1.8)	5.2 (2.0)
ASDAS	517	3.5 (0.9)	3.4 (0.9)	3.5 (0.9)	3.7 (0.9)
CRP (mg/l)	584	15.1 (20.0)	15.3 (19.0)	14.0 (20.1)	16.9 (23.4)
Elevated CRP, %	581	53.4	54.8	50.0	55.4
BASFI	555	4.1 (2.5)	3.7 (2.4)	4.2 (2.4)	5.0 (2.6)*
BASMI, median (IQR)	540	2 (1–3)	2 (1–3)	2 (1–3)	2 (1–4)
EQ-5D	540	56.2 (20.9)	58.3 (20.7)	54.7 (20.8)	51.8 (21.2)*
Peripheral arthritis, %	606	34.2	32.9	35.9	34.9
Current enthesitis, %	605	71.9	69.1	71.7	82.8
Modified MASES, median (IQR)	599	2 (0–4)	1 (0–4)	2 (0–4)	4 (2–6)*^#^
Dactylitis ever, %	616	10.2	8.3	12.2	12.9
Uveitis ever, %	555	22.2	26.4	17.4	17.7
Psoriasis ever, %	478	12.3	13.0	10.6	14.3
Current smokers, %	527	36.4	39.2	31.2	38.2
Education, high, %	594	84.0	86.8	81.9	78.0
Exercise score^†^, median (IQR)	500	2.0 (0.0–4.0)	2.0 (0.0–4.0)	2.0 (0.0–3.0)	1.5 (0.0–3.0)
On NSAIDs, %	565	92.2	92.8	91.6	91.4
On DMARDs, %	624	12.5	11.2	13.2	15.9
On steroids, %	624	9.5	10.2	7.8	10.2
First TNFi used	624				
Adalimumab, %		34.5	35.8	36.3	25.0
Certolizumab, %		<0.1	0.3	1.0	0.0
Etanercept, %		26.7	24.4	28.9	28.4
Golimumab, %		16.9	17.2	14.2	21.6
Infliximab, %		21.9	22.3	19.6	25.0

Except where indicated otherwise, values are the mean (SD)

Significance levels of two-group comparisons are Bonferroni-corrected: *p < 0.015 compared with patients of normal weight; ^#^ *p* <0 .015 compared with patients who are overweight

^†^Exercise score refers to the number of exercise sessions per week

Normal weight = BMI 18.5–25; overweight = BMI 25–30; obese = BMI >30

*ASDAS* Ankylosing Spondylitis Disease Activity Score, *BASDAI* Bath Ankylosing Spondylitis Disease Activity Index, *BASFI* Bath Ankylosing Spondylitis Functional Index, *BASMI* Bath Ankylosing Spondylitis Metrology Index, *BMI* body mass index, *CRP* C-reactive peptide, *DMARDs* disease-modifying antirheumatic drugs, *EQ-5D* EuroQol 5-domain, *GA* global assessment, *HLA-B27* human leucocyte antigen-B27, *IQR* interquartile range, *MASES* Maastricht Ankylosing Spondylitis Enthesitis Score (modification refers to the inclusion of the plantar fascia in the count), *mNYc* modified New York criteria, *NSAIDs* nonsteroidal anti-inflammatory drugs, *TNFi* tumor necrosis factor inhibitor

**Fig. 1:**
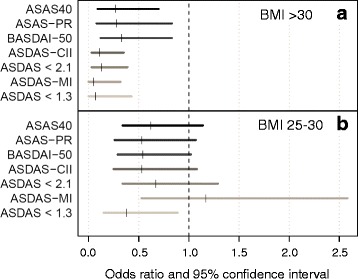
Impact of obesity (**a**) and overweight status (**b**) on different outcomes after 1 year of treatment with a first TNFi in multivariable analyses. Summarized results from different multivariable models with the same covariates as used in Model 1 in Table 3. *ASAS40* 40% improvement according to the Assessment in SpondyloArthritis International Society criteria, *ASAS-PR* partial remission criteria according to ASAS, *ASDAS* Ankylosing Spondylitis Disease Activity Score, *BASDAI-50* 50% improvement in the Bath Ankylosing Spondylitis Disease Activity Index, *BMI* body mass index, *CII* clinically important improvement, *MI* major improvement

Data on disease activity at 1 year to assess at least one of the predefined validated response criteria was available in 531 patients (85%). An ASAS40 response was reached by 44%, 34%, and 29% of patients of normal weight, overweight, and obesity, respectively (overall *p* = 0.02; Table 2). Lower response rates for overweight and obese patients were also demonstrated for the other outcomes assessed (Table 2). Evaluation of crude ASAS40 responses following stratification for treatment with infliximab versus treatment with TNFi other than infliximab revealed similar response rates in infliximab patients (42%, 36%, and 44% of patients of normal weight, overweight and obesity, respectively; overall *p* = 0.83), but not in patients treated with other TNFi (Table 2). Direct comparisons between overweight patients and normal weight patients, as well as obesity and normal weight in unadjusted analyses are shown in Additional file 3 (Table S1). Adjusted logistic regression models were fitted in patients with available data regarding known predictors of response to TNFi (*n* = 259 for the ASAS40 response). A lower ASAS40 response was found in adjusted analyses for overweight patients vs. normal BMI patients as well as for obese patients vs. patients with normal BMI, reaching statistical significance only in the latter (odds ratio (OR) 0.62, 95% confidence interval (CI) 0.24–1.14 and OR 0.27, 95% CI 0.09–0.70, respectively; Model 1 in Table 3). Significantly lower improvement criteria and status scores were also found in obese patients vs. patients with normal BMI for all the other outcomes assessed (Fig. 1a). The achievement of ASDAS improvement and status scores were particularly impaired in obesity (OR <0.15 for all four ASDAS outcomes). While lower response rates were also found for overweight patients in comparison to those with normal BMI, the differences did not reach statistical significance (Fig. 1b).

**Table 2:** Crude response rates at 1 year of treatment with a first TNF inhibitor after stratification for different BMI categories

		BMI category			
Outcome	*n =* 531	Normal *n* = 282	Overweight *n* = 178	Obese *n* = 71	*p*
ASAS40	494	44%	34%	29%	0.02
ASAS40 TNFi other than INF	383	45%	34%	24%	0.008
ASAS40 TNFi: INF	111	42%	36%	44%	0.83
ASAS partial remission	531	39%	24%	17%	<0.001
BASDAI-50	488	48%	40%	33%	0.06
ASDAS improvement ≥1.1	423	59%	46%	37%	0.003
ASDAS <2.1	468	56%	41%	25%	<0.001
ASDAS improvement ≥2	423	25%	25%	13%	0.14
ASDAS <1.3	468	29%	15%	10%	<0.001

Normal weight = BMI 18.5–25; overweight = BMI 25–30; obese = BMI >30

*ASAS* Assessment in SpondyloArthritis International Society, *ASAS40* 40% improvement according to ASAS, *ASDAS* Ankylosing Spondylitis Disease Activity Score, *BASDAI-50* 50% improvement in Bath Ankylosing Spondylitis Disease Activity Index, *BMI* body mass index, *INF* infliximab, *TNFi* tumor necrosis factor inhibitor

**Table 3:** Multiple adjusted analysis of ASAS40 response in different BMI categories at 1 year of treatment with a first TNF inhibitor

	Model 1			Model 2		
Variable	OR	95% CI	*p*	OR	95% CI	*p*
Obese (ref: normal BMI)	0.27	0.09–0.70	0.01	0.18	0.05–0.59	0.008
Overweight (ref: normal BMI)	0.62	0.24–1.14	0.13	0.66	0.34–1.30	0.23
Age	1.01	0.99–1.04	0.32	1.01	0.98–1.04	0.36
HLA-B27	1.31	0.65–2.68	0.46	1.35	0.67–2.79	0.41
Male sex	2.41	1.28–4.66	0.007	2.57	1.35–5.01	0.005
nr-axSpA (ref: AS)	0.38	0.18–0.78	0.009	0.38	0.18–0.78	0.01
Current smoking yes vs. no	0.65	0.36–1.18	0.16	0.64	0.35–1.16	0.15
Exercise (ref: no exercise)	0.89	0.50–1.58	0.69	0.83	0.46–1.50	0.54
BASDAI	1.10	0.95–1.27	0.19	1.10	0.95–1.27	0.20
BASMI	0.76	0.63–0.90	0.002	0.75	0.63–0.89	0.002
Elevated CRP	1.69	0.94–3.08	0.08	1.65	0.91–3.03	0.10
Enthesitis	1.34	0.72; 2.52	0.36	1.29	0.69; 2.43	0.43
DMARDs	1.17	0.51; 2.71	0.71	1.14	0.48; 2.67	0.76
NSAIDs	1.21	0.39; 4.24	0.75	1.13	0.35; 4.07	0.84
Infliximab (ref: other TNFi)				0.66	0.26–1.65	0.37
Obese with infliximab (ref: other TNFi)				3.55	0.41–30.1	0.24
Overweight with infliximab (ref: other TNFi)				0.73	0.15–3.26	0.68

Analysis performed in 259 patients

*AS* Ankylosing Spondylitis, *BASDAI* Bath Ankylosing Spondylitis Disease Activity Index, *BASMI* Bath Ankylosing Spondylitis Mobility Index, *BMI* body mass index, *CI* confidence interval, *CRP* C-reactive peptide, *DMARDs* disease-modifying antirheumatic drugs, *HLA-B27* human leucocyte antigen-B27, *nr-axSpA* nonradiographic axial spondyloarthritis. *NSAIDs* nonsteroidal anti-inflammatory drugs, *OR* odds ratio, *ref* reference, *TNFi* tumor necrosis factor inhibitor

To analyze whether missing covariate data affected these results, unadjusted analyses were also performed for the subpopulation of patients with complete covariate values. Response rates in this subgroup of patients were comparable to the outcomes of the whole population (Additional file 4: Table S2).

In a sensitivity analysis of the adjusted ASAS40 response, we included infliximab as a covariate as well as interaction terms between infliximab administration and the different BMI groups in the model in order to account for the fact that infliximab is dosed in a weight-dependent manner (Model 2 in Table 3). Although no statistical significance could be demonstrated for these interactions, the results suggest a trend for higher ASAS40 responses in obese patients treated with infliximab versus obese patients treated with other anti-TNF agents (OR 3.55, 95% CI 0.41–30.1; *p* = 0.24). This trend was not found in overweight patients. However, in the overall population, a trend for lower ASAS40 responses was demonstrated in patients treated with infliximab versus patients treated with other anti-TNF agents (OR 0.66, 95% CI 0.26–1.65; *p* = 0.37).

**Fig. 2:**
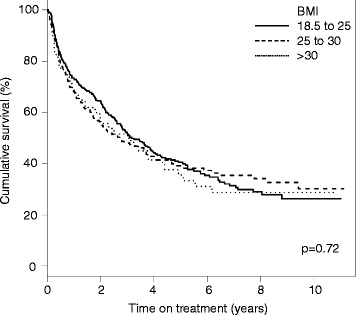
Drug survival of the first TNFi, stratified by body mass index (*BMI*) group

With regard to drug maintenance, Kaplan-Meyer plots demonstrated a comparable TNFi retention in the different BMI groups (log-rank test *p* = 0.72; Fig. 2). The result was confirmed in a multiple adjusted Cox proportional hazards model (*n* = 356; Table 4). The hazard ratio (HR) for discontinuing the first TNFi in obese patients in comparison to individuals with normal BMI was 1.01 (95% CI 0.63–1.65; *p* = 0.95). The respective HR for discontinuation in overweight vs. normal weight patients was 0.98 (95% CI 0.79-1.38; *p* = 0.92).

**Table 4:** Multiple adjusted Cox proportional hazards model for analysis of drug discontinuation of a first TNF inhibitor in different BMI categories

Variable	HR	95% CI	*p*
Obese (ref: normal BMI)	1.01	0.63–1.65	0.95
Overweight (ref: normal BMI)	0.98	0.79–1.38	0.92
Age	1.00	0.98–1.01	0.73
HLA-B27 positivity	0.86	0.60–1.25	0.43
Male sex	0.68	0.49–0.96	0.03
nr-axSpA (ref: AS)	1.47	1.01–2.14	0.04
Current smoking yes vs. no	0.92	0.66–1.28	0.61
Exercise yes vs. no	0.82	0.60–1.12	0.21
BASDAI	1.04	0.96–1.13	0.35
BASMI	1.07	0.97–1.18	0.17
Elevated CRP	0.71	0.51–0.98	0.04
Enthesitis	0.91	0.64–1.30	0.61
DMARDs	0.56	0.34–0.93	0.02
NSAIDs	1.00	0.55–1.83	0.99

Analysis performed in 343 patients

*AS* Ankylosing Spondylitis, *BASDAI* Bath Ankylosing Spondylitis Disease Activity Index, *BASMI* Bath Ankylosing Spondylitis Mobility Index, *BMI* body mass index, *CI* confidence interval, *CRP* C-reactive peptide, *DMARDs* disease-modifying antirheumatic drugs, *HLA-B27* human leucocyte antigen-B27, *HR* hazard ratio, *nr-axSpA* nonradiographic axial spondyloarthritis. *NSAIDs* nonsteroidal anti-inflammatory drugs, *ref* reference, *TNF* tumor necrosis factor

## Discussion

Up to 50% of patients with axSpA initiating a first TNFi in the SCQM cohort presented with a BMI above the normal range, and 14% were obese. Being overweight and particularly obese was associated with an impaired response to TNFi, as assessed by a multitude of validated outcomes. While being overweight decreased the odds of achieving an ASAS40 response upon TNF inhibition by about 30%, the odds were decreased by 70% in obese patients. Achievement of ASDAS improvement and status scores was even more severely impaired in obese patients. Importantly, BMI has been shown to not affect the determination of the ASDAS in a recent analysis of the SPACE cohort [14]. A previous study had suggested that the negative impact of high BMI on treatment response might be more important in patients treated with infliximab in comparison to other anti-TNF drugs [8]. This finding is intriguing as infliximab is the only anti-TNF agent administered in a weight-dependent manner. We hereby demonstrate in a larger cohort of patients with axSpA that an impaired response to TNFi is also found for the other anti-TNF agents. The proportion of patients treated with infliximab in our study was similar in all BMI categories, indicating that rheumatologists in Switzerland do not preferentially use infliximab for axSpA treatment in overweight and obese patients. We found a trend for a better ASAS40 response in obese patients treated with infliximab in comparison to treatment with other anti-TNF agents. Our results suggest potential underdosing of subcutaneous TNFi in patients with obesity. An alternative, though not mutually exclusive, hypothesis put forward to explain the impaired treatment outcomes in obese patients with inflammatory rheumatic diseases is the amplified production of proinflammatory adipokines by fat tissue [15, 16]. Obese patients with psoriatic arthritis have indeed been found to have a lower probability of achieving a sustained minimal disease activity state independently of the use of DMARDs and biologics [6].

We demonstrate that drug retention was similar irrespective of BMI category, indicating that in the absence of alternative treatment options some patients remain on a particular anti-TNF agent despite an inadequate treatment response. This is in contrast to current treatment recommendations, which stipulate to consider continuing treatment only in patients with an ASDAS and/or BASDAI improvement of ≥1.1 and ≥2, respectively [17].

A positive effect of weight loss induced by hypocaloric diet on TNFi response has been demonstrated in psoriatic arthritis [18], indicating that obesity is a manageable risk factor for the low performance of TNFi in inflammatory rheumatic diseases. Advice on the management of obesity [19, 20] might thus not only reduce cardiovascular risk but also improve therapeutic response. This might be particularly important for TNFi usage which has been shown to be associated with significant weight gain in spondyloarthritis, mostly due to an increase in android fat mass [21].

We utilized BMI as a proxy for overweight status and obesity due to feasibility issues in this large cohort. This might be suboptimal, given the individual variability in the relationship between BMI and body fat [19]. Additional data using direct quantification of fat mass in smaller cohorts of patients with axSpA might help in elucidating the pathophysiology of impaired treatment responses in patients with high BMI.

Some limitations are incurred given the observational character of our real-life cohort and, in particular, missing covariate data in the adjusted analyses. Crude response rates in the subpopulation of patients with complete data were comparable to the values in the unadjusted analyses with all available patients.

## Conclusion

In conclusion, obesity is associated with significantly lower response rates to TNFi in patients with axSpA.

## Additional files

Additional file 1: Figure S1. Schematic representation of height correction for kyphosis using the occiput-to-wall distance. (DOC 90 kb)

Additional file 2: Figure S2. Body height before and after correction for kyphosis by using the occiput-to-wall distance. (DOC 118 kb)

Additional file 3: Table S1. Impact of obesity and overweight status on different outcomes after 1 year of treatment with a first TNFi in unadjusted analyses. (DOC 34 kb)

Additional file 4: Table S2. Crude response rates at 1 year of treatment with a first TNFi after stratification for different BMI categories for the population with complete covariate data in multivariable analyses. (DOC 37 kb)

## Abbreviations

AS: Ankylosing spondylitis
ASAS: Assessment in SpondyloArthritis International Society
ASAS-PR: Partial remission criteria of the Assessment of Spondyloarthritis International society
ASDAS: Ankylosing Spondylitis Disease Activity Score
axSpA: Axial spondyloarthritis
BASDAI: Bath Ankylosing Spondylitis Disease Activity Index
BASDAI-50: 50% improvement in BASDAI
BASFI: Bath Ankylosing Spondylitis Functional Index
BASMI: Bath Ankylosing Spondylitis Mobility Index
BMI: Body mass index
CI: Confidence interval
CRP: C-reactive protein
DMARD: Disease modifying antirheumatic drug
HLA-B27: Human leucocyte antigen-B27
HR: Hazard ratio
MASES: Maastricht Ankylosing Spondylitis Enthesitis Score
nr-axSpA: Nonradiographic axial spondyloarthritis
OR: Odds ratio
SCQM: Swiss Clinical Quality Management
TNFi: Tumor necrosis factor inhibitors
