# Supplementary material for: Impact of obesity on the response to tumor necrosis factor inhibitors in axial spondyloarthritis
Source: Arthritis Res Ther. 2017 Jul 19;19:164. doi: 10.1186/s13075-017-1372-3 (PMC5518107; doi:10.1186/s13075-017-1372-3)

**Figure S1.** Schematic representation of height correction for kyphosis using the occiput-to-wall distance


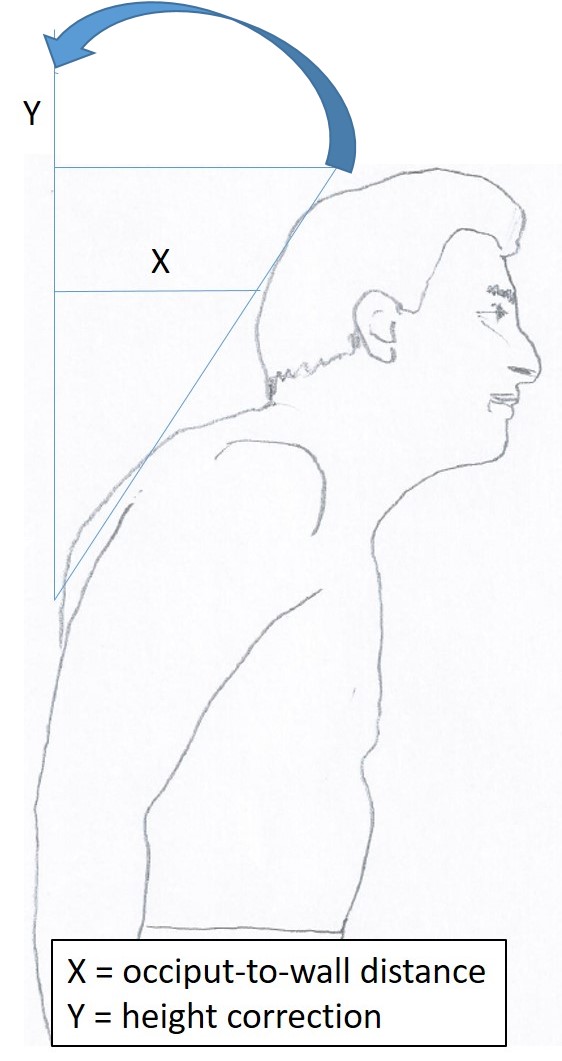

Supplement: Supplementary file 1 — Schematic representation of height correction for kyphosis using the occiput-to-wall distance. (DOC 90 kb) [file 13075_2017_1372_MOESM1_ESM.doc]
