# Supplementary material for: Impact of obesity on the response to tumor necrosis factor inhibitors in axial spondyloarthritis
Source: Arthritis Res Ther. 2017 Jul 19;19:164. doi: 10.1186/s13075-017-1372-3 (PMC5518107; doi:10.1186/s13075-017-1372-3)
Supplement: Supplementary file 3 — Impact of obesity and overweight status on different outcomes after 1 year of treatment with a first TNFi in unadjusted analyses. (DOC 34 kb) [file 13075_2017_1372_MOESM3_ESM.doc]

**Table S1. Impact of obesity and overweight status on different outcomes after 1 year of treatment with a first TNFi in unadjusted analyses**

|  |  | **BMI category** | | | | | |
| --- | --- | --- | --- | --- | --- | --- | --- |
|  |  | **Obese vs. normal BMI** | | | **Overweight vs. normal BMI** | | |
| **Outcome** | **N** | **OR** | **95% CI** | **P** | **OR** | **95% CI** | **P** |
| **ASAS40** | 494 | 0.51 | 0.28-0.890 | 0.02 | 0.65 | 0.43-0.97 | 0.03 |
| **ASAS partial remission** | 531 | 0.32 | 0.16-0.61 | <0.001 | 0.51 | 0.33-0.76 | 0.001 |
| **BASDAI-50** | 488 | 0.54 | 0.30.0.96 | 0.04 | 0.71 | 0.48-1.06 | 0.10 |
| **ASDAS improvement ≥1.1** | 423 | 0.40 | 0.22-0.74 | 0.004 | 0.58 | 0.38-0.88 | 0.01 |
| **ASDAS <2.1** | 468 | 0.26 | 0.13-0.48 | <0.001 | 0.55 | 0.37-0.82 | 0.004 |
| **ASDAS improvement ≥2** | 423 | 0.44 | 0.17-0.97 | 0.06 | 0.98 | 0.60-1.59 | 0.95 |
| **ASDAS <1.3** | 468 | 0.26 | 0.10-0.60 | 0.003 | 0.44 | 0.26-0.72 | 0.002 |

ASAS40 = 40% improvement according to the Assessment in SpondyloArthritis International Society criteria; ASDAS = Ankylosing Spondylitis Disease Activity Score; BMI = Body Mass Index; OR = odds ratio; 95% CI = 95% confidence interval. TNFi = Tumor Necrosis Factor inhibitor.
