# Supplementary material for: Impact of obesity on the response to tumor necrosis factor inhibitors in axial spondyloarthritis
Source: Arthritis Res Ther. 2017 Jul 19;19:164. doi: 10.1186/s13075-017-1372-3 (PMC5518107; doi:10.1186/s13075-017-1372-3)
Supplement: Supplementary file 4 — Crude response rates at 1 year of treatment with a first TNFi after stratification for different BMI categories for the population with complete covariate data in multivariable analyses. (DOC 37 kb) [file 13075_2017_1372_MOESM4_ESM.doc]

**Table S2. Crude response rates at 1 year of treatment with a first TNFi after stratification for different BMI categories for the population with complete covariate data in multivariable analyses**

|  | **BMI category** | | |  |
| --- | --- | --- | --- | --- |
| **Outcome** | **Normal**  **N=135** | **Overweight**  **N=80** | **Obese**  **N=26** | **p** |
| **ASAS40** | 45 | 42 | 15 | 0.01 |
| **ASAS partial remission** | 39 | 31 | 19 | 0.13 |
| **BASDAI-50** | 52 | 46 | 27 | 0.07 |
| **ASDAS improvement ≥1.1** | 63 | 42 | 27 | 0.003 |
| **ASDAS <2.1** | 56 | 50 | 19 | 0.002 |
| **ASDAS improvement ≥2** | 26 | 29 | 4 | 0.02 |
| **ASDAS <1.3** | 25 | 15 | 4 | 0.02 |

Except where indicated otherwise, values are the %. ASAS = Assessment in SpondyloArthritis international Society; ASAS40 = 40% improvement according to ASAS; ASDAS = Ankylosing Spondylitis Disease Activity Score; BMI = Body Mass Index. Normal weight = BMI 18.5 to 25; Overweight = BMI >25 to 30; Obese = BMI >30.
